# Biometry

**Published:** 1875-07-01

**Authors:** Moreau Morris

**Affiliations:** New York


### BIOMETRY.

By Moreau Morris, M.D., New York.

*From the Sanitarian, July, 1875.*

FROM the earliest history of medicine there has always been recognized an indefinable something inherent in the human system, varying in degree and force—this has been variously designated the "tenacity of life," the "tolerance of disease," the "natural vigor of constitution," the "vis medicatrix naturæ,"—by which some individuals seem to be able to endure and pass successfully through the most serious of maladies, or the most severe injuries, without succumbing. Many instances might be enumerated in illustration, but every practitioner will readily recognize such examples in his own experience. How often persons have recovered after injuries, gunshot wounds, and exhaustive diseases, who, at the time, to all human foresight, seemed beyond recovery.

There is some inherent principle which mysteriously sustains life through these severe onslaughts. We must recognize a "vis preservatrix" and a "vis a tergo." What this force consists of neither Anatomy, Physiology, Pathology, Microscopy, nor Chemistry has been able to elucidate. We know that man inherits vital properties which are in force from conception to death; that his various components are endowed with life-times of variable duration; that, like other living things, some parts decay and perish before others in regular succession. One day we see the plants beneath our feet spring up, throw out their green leaves and budding flowers, all endowed apparently with vigorous blooming life; and in a few months, or perhaps days, their flowers, leaves, and stalks fade, wither and die. These are but prototypes of man. He springs up, flourishes for a time in full vigor, and one by one, his secerning organs fail, until at last his physical entity ceases. The vital property has ceased to carry on its secretive power in one organ after another, until it can no longer sustain life. It is not within human ken to describe this vital property.

God breathed into our bodies life, which proceeds under the various laws of our being, so long as they are not violated, until the human machine wears out. It is within our power to cut it short, but not to prolong it beyond its natural inheritance. We can study its processes, observe the laws which govern it, judge of its force approximately, see its manifestations, and *estimate its probable period.* There are certain *uniform indications* by which we may judge of man's *probable life-time.* Some are *endowed* with short life, some with a probably long and healthy life.

Inherited tendencies, habits of living, occupations, observance of sanitary law, and residence—all have their direct bearings upon the question of longevity. Acute diseases, accidents, etc., have their life-shortening influences. All of these must be studied in their various relations to length of life.

The study of biometry is comparatively of but recent date. Like every other science, its study involves labor and care; statistics are to be collected and compared, its rules and laws elucidated and fixed, to make its practical application of value. When these laws become understood, their application is readily recognized. In medicine, in life insurance, in business, in social life, in a higher elevation of mankind generally, both physically and morally, the application of the science of biometry will be found invaluable. The laws of natural selection, by which physical perfection may be attained, will find in its exposition the true guide-posts by which to accomplish that much-desired result. Intuitively we all apply its principles, even without, perhaps, being able to analyze the reasons for our judgment. The physician, by observation and long force of habit, is constantly applying its fundamental truths. He sees nature asserting and exhibiting wonderful endurance and adaptation under the most adverse circumstances, yet he is unable to define or explain the reasons.

In every-day life we constantly apply its principles in our intuitive estimation of our fellows; we judge of men's qualities or adaptation for certain kinds of business without system or explainable method.

To Dr. T. S. Lambert, more than any other man, belongs the credit of having studied and reduced to a scientific basis the development and application of this instructive and interesting science. During many years of close application and observation he has fortified its truth by thousands of examples, and so simplified its practical application to the business of life-insurance, that its laws have become the fixed data in estimating the probabilities of life's period; and as this business, when scientifically and successfully transacted, very largely depends upon a correct estimate and judgment of the probable length of any proposed life, as a matter of security and equity, its application in this direction has already, in the company with which he is connected, reduced the hazard of the business to one of great certainty.*

The laws of biometry are abundantly illustrated by heredity. The histological characteristics of persons when studied under these laws present the most convincing proofs of the status of biometry as a true science. In the examination of the ancestral histories of thousands of individuals, the deductions therefrom establish the fact that certain measurements can be relied upon almost infallibly, by which to read backward from the person the life characteristics of the ancestry, and hence inversely to determine the individual's life probabilities. So, when we find a person presenting these general measures in due proportion, we may judge, almost invariably, of his powers of resistance or natural vitality. If so be he is descended from a healthy, long-lived stock of both parents, almost without exception it will be found as a rule that he is both healthy and long-lived, able to endure much hardship, resist grave maladies, and to recover from the most serious injuries and great nervous shocks.

* The former method of basing the cost upon the general average death rate of all mankind, as deduced from various experience tables, has, in that company at least, been discarded, and the results already accomplished by the application of this science to the problem has created the greatest astonishment in the life insurance world. Death ratios, from having been ten to twelve per thousand among selected lives, are reduced by this method with almost absolute certainty to less than four per thousand; hence the direct pecuniary benefit to the world, as respects life insurance, is more than three-fold as regards the proper cost to such risks.

Again, it is found from observation that where there has been long and vigorous ancestral stock upon one side, with perhaps short life engrafted from the other, such person will arrive at a period of partial decline, with ill health, and subsequently recover, living on and beyond this deflection, being sustained by the vitalizing, secretory influences of the longer-lived ancestor. A moment's reflection will call to mind many such instances, as when persons have remarked that at a certain period of their lives they were suffering from some special disorder, from which, after a period, they have seemingly entirely recovered and enjoyed sound, robust health.

That longevity is a resultant of heredity no one will dispute, and that it does not wholly depend upon race, climate, mode of life, or special observance of sanitary law, is also a self-evident fact. Those who have inherited it can, seemingly with impunity, almost defy all sanitary law, and yet continue to live up to and beyond the allotted limit of "three score years and ten," while those who have not inherited long life cannot by any system of life, or observance of the laws of health or process of prolongation, protract their naturally short-lived inheritance. Of course we must admit that abuse can and does shorten the lives of the naturally long-lived, and acute disease or great injuries cut them off suddenly; but the rule holds good that the naturally long-lived inheritance affords that innate power of resistance which will carry them through disaster and disease that will certainly destroy the naturally short-lived.

The probably short-lived may be equally healthy and robust, and able to endure almost as much, while that life lasts, as the longer-lived, yet it seems to be the fact and nature's law, that the period of existence has had its set limit beyond which no process of prolonging can avail to carry it beyond the allotted period. The secerning elements of the vital organs have their limits, and hence control the existence of the whole organism. We see this illustrated in almost every organ of the body; certain parts cease to perform their functions, die out; and, so long as these are not vital, life continues, although it may be in a restricted sense — as, for instance, persons become bald, or partially so, at a certain age; they say the same occurred in their ancestors at about the same age; others find their digestive powers failing and remark the same thing as having occurred in their parents or grandparents. The secretory vitality of these parts is then seen to follow the law of heredity.

Without an inheritance of long-lived secretory powers it is in vain to expect any great degree of longevity in the descendants.

In estimating the probability of a life-time, it is entirely useless to depend upon the general average of human life. This rule holds good only as respects human life at large, and therefore we must look beyond life statistics to sum up the problem. *With the duration of individual life general average holds no command.* Ancestral longevity will not obey the general average law, but defies death in many shapes, holding on tenaciously until the machine, actually from rust and the interstitial deposits of years among its most delicate parts, wears out. * * * * * *

Dr. Lucas, in his *Traite Physiologique et Philosophique de l'Heredite Naturelle*, remarks as follows: "The *average of life* plainly depends on locality, hygiene, and civilization; but the *individual longevity* is entirely exempt from these conditions."

"Everything tends to show that long life is the result of an internal principle of vitality which privileged individuals receive at their birth. It is so deeply imprinted in their nature as to *make itself apparent in every part of their organization.*"*

The foregoing statement of Dr. Lucas is also quoted with emphatic approval in a recent work on Heredity, by Ribot, of whom Dr. Lambert

* The italics are ours.

remarks that he "may be justly regarded as the ablest of European writers upon this subject."

This interesting and practically important idea of the different lengths of life is well illustrated in the hair glands on different heads, not only, but on the same head. Some hair glands inherit a life of ninety years, while their fellows terminate their inherited longevity at twenty years or under. * * * How often we see baldness follow ancestry, even in quantity and position; and the question cannot be avoided, Does not analogy legitimately argue that a similar condition should be expected in every other organ of the body possessing a community of glands?

It is not enough that we analyze the appearance of patients, so that we can discern which *organs* are affected; but we should be able to recognize to what *extent* they are impaired, how large a *portion* of them has reached the natural terminus of the longevity belonging thereto, and which is bound to die then and there. If this portion is large enough, and belongs to a sufficiently vital organ, to commit homicide upon the other organs of the body depending for life upon the dying portions, it matters not how long-lived the other portions or the other organs may be by inheritance, they must then and there die from inanition. Marasmus is an apt illustration of a homicidal death by this method.

In such cases there will be at first a general appearance of much vigor, and a man of but little observation would be likely to prognosticate recovery, not remembering that the chain is never stronger than its weakest link. We must observe the weak spots. Then shall we find that many more deaths are produced by natural, unavoidable causes—namely, the termination of the inherited naturally short life of some organ or portions of it indispensable to the continuance of the whole, than we usually have supposed; whilst again many recover from severe attacks on account of the inherent longevity of such a proportional part of the diseased organ that there really was no danger of dying, even under the worst kind of treatment. These suggestions account for the apparent success of quacks and ignorant pretenders. * * * By instituting comparisons, or observing certain general configurations uniformly found in a very large number of individuals, it has been found that certain universal conditions pertain to the long-lived and to the short-lived exclusively. These are found in the size, shape, proportion, color, and capacity of all parts of the body.

Thus we can compare persons descended from long-lived with those from short-lived ancestors, and notice the differences which, as a practical fact, are *found to be well-defined;* for example, the following: the comparative size and shape of the head; the colors of its external components, as hair, beard, eye-brows, eyes, shape and size of nose, lips, chin and features in general, and their comparative relative measures; the trunk with its relative proportions. It may be here remarked that the *length* of the trunk has even a more important significance than the circumference; for when the proportion of the trunk is in excess of one-third the height of the figure, we may be assured of corresponding great life, tenacity and capacity. A comparatively long trunk gives us a form that affords room for the functions of respiration and digestion, the two most important life-sustaining functions of the whole organism.

Given good respiratory capacity and good digestive apparatus, may we not prognosticate a healthy, vigorous constitution?

In looking over these indicative points, even in the sick, we need also to inquire into ancestral characteristics. What has been, not the average, but the special duration of the antecedent lives of the individual's progenitors? What were their peculiar diseases, family diseases, so called; and of what diseases, and at what ages, did they die, if dead?

Here lies the clue to the factors of the disease under observation in any given case.

By observing and applying the laws of biometry in the treatment of disease, the medical man places himself in the front rank of the benefactors of mankind, and he is also thereby enabled the better to apply the great laws of hygiene. Observing the temperament, the tendencies to some special form of disease, the predispositions, he is qualified to extend his warning advice regarding occupation, residence, and habits of life, and to suggest at what periods of life may be expected certain ailments, and the necessary precautions to avoid, if possible, their worst effects.

Thus, in applying the laws of biometry, we may not only be useful to our fellow-man in curing disease, but also as conservators by our forewarnings.

